# Biosynthesis and engineering of kaempferol in *Saccharomyces cerevisiae*

**DOI:** 10.1186/s12934-017-0774-x

**Published:** 2017-09-26

**Authors:** Lijin Duan, Wentao Ding, Xiaonan Liu, Xiaozhi Cheng, Jing Cai, Erbing Hua, Huifeng Jiang

**Affiliations:** 1 Key Laboratory of Industrial Microbiology, College of Biotechnology, Tianjin University of Science & Technology, Tianjin, China; 2 Key Laboratory of Systems Microbial Biotechnology, Tianjin Institute of Industrial Biotechnology, Chinese Academy of Sciences, Tianjin, China; 3 University of Chinese Academy of Sciences, Beijing, China; 4 State Key Laboratory of Quality Research in Chinese Medicine, Institute of Chinese Medical Sciences, University of Macau, Macau, China

**Keywords:** Kaempferol, Flavonol synthase, Acetyl-CoA, *Saccharomyces cerevisiae*

## Abstract

**Background:**

Kaempferol is a flavonol with broad bioactivity of anti-oxidant, anti-cancer, anti-diabetic, anti-microbial, cardio-protective and anti-asthma. Microbial synthesis of kaempferol is a promising strategy because of the low content in primary plant source.

**Methods:**

In this study, the biosynthesis pathway of kaempferol was constructed in the budding yeast *Saccharomyces cerevisiae* to produce kaempferol de novo, and several biological measures were taken for high production.

**Results:**

Firstly, a high efficient flavonol synthases (FLS) from *Populus deltoides* was introduced into the biosynthetic pathway of kaempferol. Secondly, a *S. cerevisiae* recombinant was constructed for de novo synthesis of kaempferol, which generated about 6.97 mg/L kaempferol from glucose. To further promote kaempferol production, the acetyl-CoA biosynthetic pathway was overexpressed and *p*-coumarate was supplied as substrate, which improved kaempferol titer by about 23 and 120%, respectively. Finally, a fed-batch process was developed for better kaempferol fermentation performance, and the production reached 66.29 mg/L in 40 h.

**Conclusions:**

The titer of kaempferol in our engineered yeast is 2.5 times of the highest reported titer. Our study provides a possible strategy to produce kaempferol using microbial cell factory.

**Electronic supplementary material:**

The online version of this article (doi:10.1186/s12934-017-0774-x) contains supplementary material, which is available to authorized users.

## Background

Kaempferol is a polyphenol anti-oxidant found in many edible plants, which have been commonly used in traditional medicine (e.g. *Ginkgo biloba*, Tilia spp., *Equisetum* spp., *Moringa oleifera*, Sophora japonica and propolis) [1, 2]. Dietary kaempferol has attracted extensive attention because of the beneficial effects on human health, including anti-oxidant, anti-inflammatory, anti-microbial, anti-cancer, cardio-protective, neuro-protective, anti-diabetic, anti-osteoporotic, estrogenic, anti-estrogenic, anxiolytic, analgesic and anti-allergic activities [1–4]. Interestingly, although kaempferol inhibits cancer cell growth and induces cancer cell apoptosis, it appears to preserve or protect normal cell viability [2]. However, the content of kaempferol in plants is very low, which results in high cost for kaempferol production from traditional plant extraction [5, 6]. Thus, it is a promising alternative strategy to produce kaempferol using microbial cell factory.

Biosynthetic pathway of kaempferol has been identified in plant [5]. At the initial stage, phenylalanine is converted into *p*-coumaryl-CoA by phenylalanine ammonia lyase (PAL), cinnamic acid 4-hydroxylase (C4H) and 4-coumaric acid ligase (4CL). Then, naringenin is generated by condensation reaction between one molecular *p*-coumaryl-CoA and three molecules of malonyl-CoA by chalcone synthesis (CHS) and chalcone isomerase (CHI). At last, the naringenin is converted into kaempferol via dihydrokaempferol by flavanone 3β-hydroxylase (F3H) and flavonol synthase (FLS). Although kaempferol has been successfully synthesized by engineered microbes, the titer of kaempferol is still lower than many other microbial produced flavonoids, such as naringenin and eriodictyol [7–11]. One of the possible causes is that the carbon flux toward precursors is usually not enough [12]. Engineering metabolic pathway toward acetyl-CoA or malonyl-CoA would partially solve this problem [13–15]. In addition, the low efficiency of key enzymes in kaempferol biosynthetic pathway could also cause this issue. It has been reported that although the precursor naringenin was sufficiently supplied, the recombinant cell factory still produced kaempferol at a very low level [10].

In this study, we proposed to construct a yeast cell factory to improve kaempferol production. Firstly, we would screen higher efficient FLS from different plants to improve kaempferol conversion rate from the precursor naringenin. Secondly, the selected FLS would be combined with other pathway genes to build a microbial cell factory for de novo kaempferol synthesis. Moreover the acetyl-CoA and malonyl-CoA pathways would be engineered to further improve kaempferol biosynthesis. *p*-Coumarate would be also supplemented as substrate to improve the precursors supply. Finally, the fermentation condition would be optimized for better kaempferol production.

## Results and discussion

### Biosynthesis of kaempferol from naringenin

**Fig. 1:**
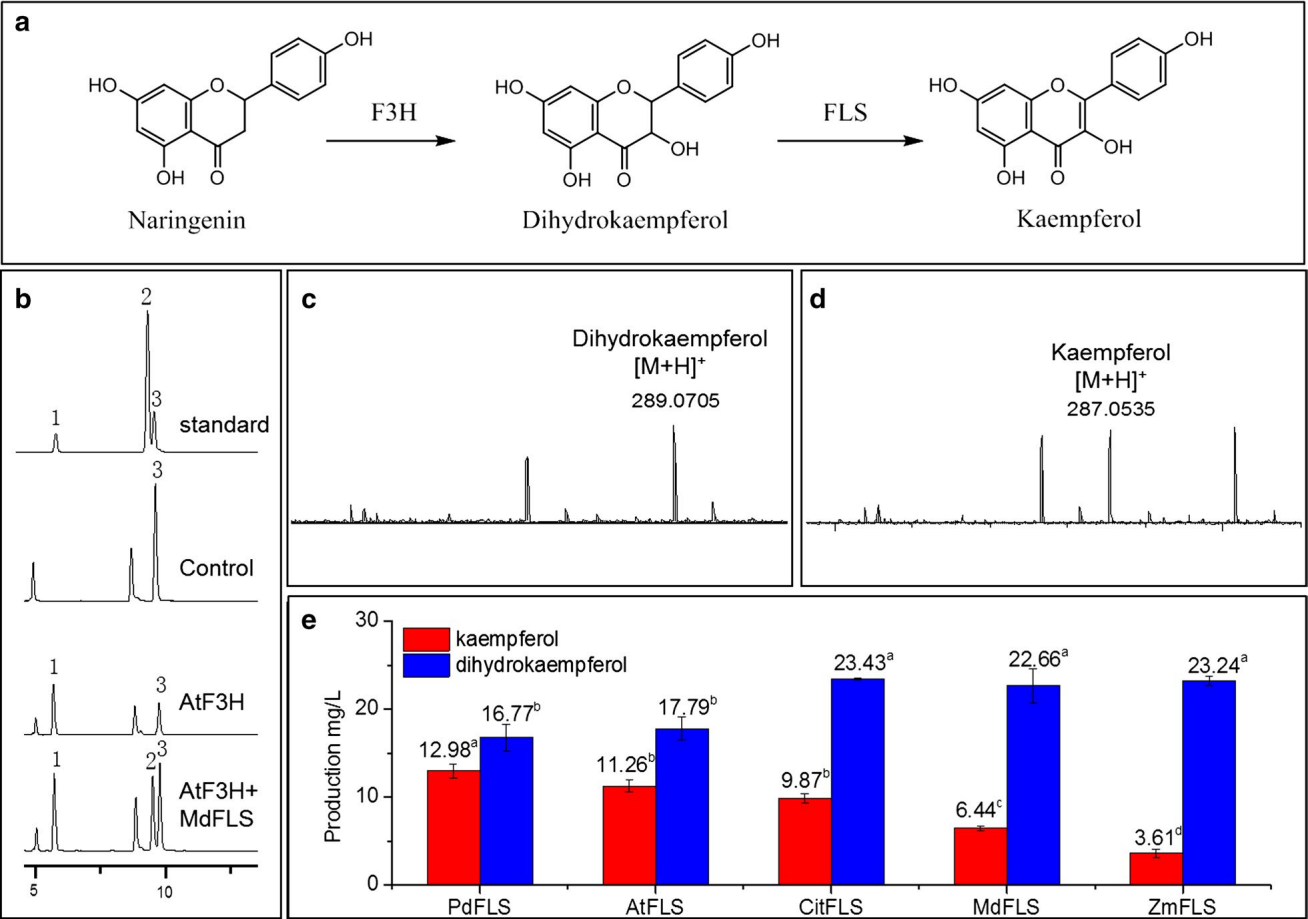
Functional identification of two key enzymes of F3H and FLS in kaempferol biosynthetic pathway. **a** The biosynthetic pathway from naringenin to kaempferol. Naringenin is converted into dihydrokaempferol with flavanone 3β-hydroxylase (F3H), and dihydrokaempferol is converted into kaempferol via flavonol synthase (FLS). **b** HPLC analysis of the product in whole cell catalysis with overexpression of F3H and FLS in yeast. Chromatogram data were collected at 335 nm. Peak 1, (+)-dihydrokaempferol; peak 2, kaempferol; peak 3, (2S)-naringenin. (2S)-naringenin was the substrate and was detected in the wild type W3-Y22. **c** LC–MS analysis of dihydrokaempferol produced by W3-*AtF3H* in whole cell catalysis. **d** LC–MS analysis of kaempferol produced by W3-*MdFLS* in whole cell catalysis. **e** Production of kaempferol and dihydrokaempferol of recombinant yeast strain expressing FLS from five plant species. Recombinant yeast strain expressing *AtF3H* and FLS from different species were fermented. Data are shown as average in triplicate assays. Error bar shows standard division. Different superscript letter indicates significant difference in Tukey analysis (α = 0.05)

Despite the significant pharmaceutical effect of kaempferol, the low content in plant or engineered microbes has restricted its application. We firstly attempted to construct the kaempferol pathway in *S. cerevisiae* from naringenin, a common intermediate of flavonoid biosynthesis. Naringenin is converted into kaempferol via dihydrokaempferol, catalyzed with flavanone 3β-hydroxylase (F3H) and flavonol synthase (FLS) (Fig. 1a). In this research, F3H from *Arabidopsis thaliana* [16] and FLS from *Malus domestica* [17] (encoded by *AtF3H* and *MdFLS* respectively) were expressed in *S. cerevisiae* to build a kaempferol-producing recombinant. Indeed, using (*2S*)-naringenin as substrate, the recombinant yeast expressing *AtF3H* produced (+)-dihydrokaempferol (Fig. 1b, c). On the other hand, both (+)-dihydrokaempferol and kaempferol were detected in the medium fermented by the recombinant expressing *AtF3H* and *MdFLS* (Fig. 1b, d). Moreover, the molecular weights of (+)-dihydrokaempferol and kaempferol were confirmed by liquid chromatograph–mass spectrometer (LC–MS) (Fig. 1c, d). However, when 100 mg/L initial (*2S*)-naringenin was supplemented, 23.62 mg/L (+)-dihydrokaempferol and only 6.03 mg/L kaempferol were produced through 24 h conversion. The relatively low titer of kaempferol indicated that the step from (+)-dihydrokaempferol to kaempferol is the bottleneck of kaempferol production from naringenin.

To solve this problem, we proposed to find a more efficient FLS from other plant sources. FLS genes from five plant species, including *A. thaliana*, *Citrus unshiu*, *M. domestica*, *P. deltoides* and *Zea Mays* [10, 18–21] (encoded by *AtFLS*, *CitFLS*, *MdFLS*, *PdFLS* and *ZmFLS*, respectively), were overexpressed with *AtF3H* in yeast, respectively. The viability of these FLS in kaempferol production were compared according to the final kaempferol productions in whole cell catalysis. The strain expressing *PdFLS* resulted in the highest kaempferol production (12.98 mg/L, Fig. 1e) and the lowest dihydrokaempferol accumulation (16.77 mg/L, Fig. 1e), indicating that *PdFLS* has the highest efficiency to produce kaempferol from dihydrokaempferol (Additional file 1: Figure S1A, S1B). Besides, the recombinants expressing *AtFLS* and *CitFLS* produced lower kaempferol than that expressing *PdFLS*, but significant higher than that expressing *MdFLS* and *ZmFLS* (Fig. 1e). According to the previous studies, FLS from *P. deltoides*, *A. thaliana* and *C. unshiu* performed both FLS and F3H function, which are able to use naringenin as a substrate to produce kaempferol as well as dihydrokaempferol [20–23]. However, this F3H function was not observed in FLS from *M. domestica* and *Z. Mays* [17, 18]. This could partially explain that the recombinants expressing *PdFLS*, *AtFLS* and *CitFLS* produced significant higher kaempferol than those expressing *MdFLS* and *ZmFLS*. Although the sole expression of bifunctional FLS would enable the recombinant to convert naringenin into kaempferol, the co-expression with F3H would promote flavonol titer and has been widely adopted in the previous studies [9, 24]. Considering the co-expression of *PdFLS* and *AtF3H* resulted in the highest kaempferol production from naringenin, this gene cluster was retained for further kaempferol biosynthesis.

### Construction of de novo synthetic pathway of kaempferol

**Fig. 2:**
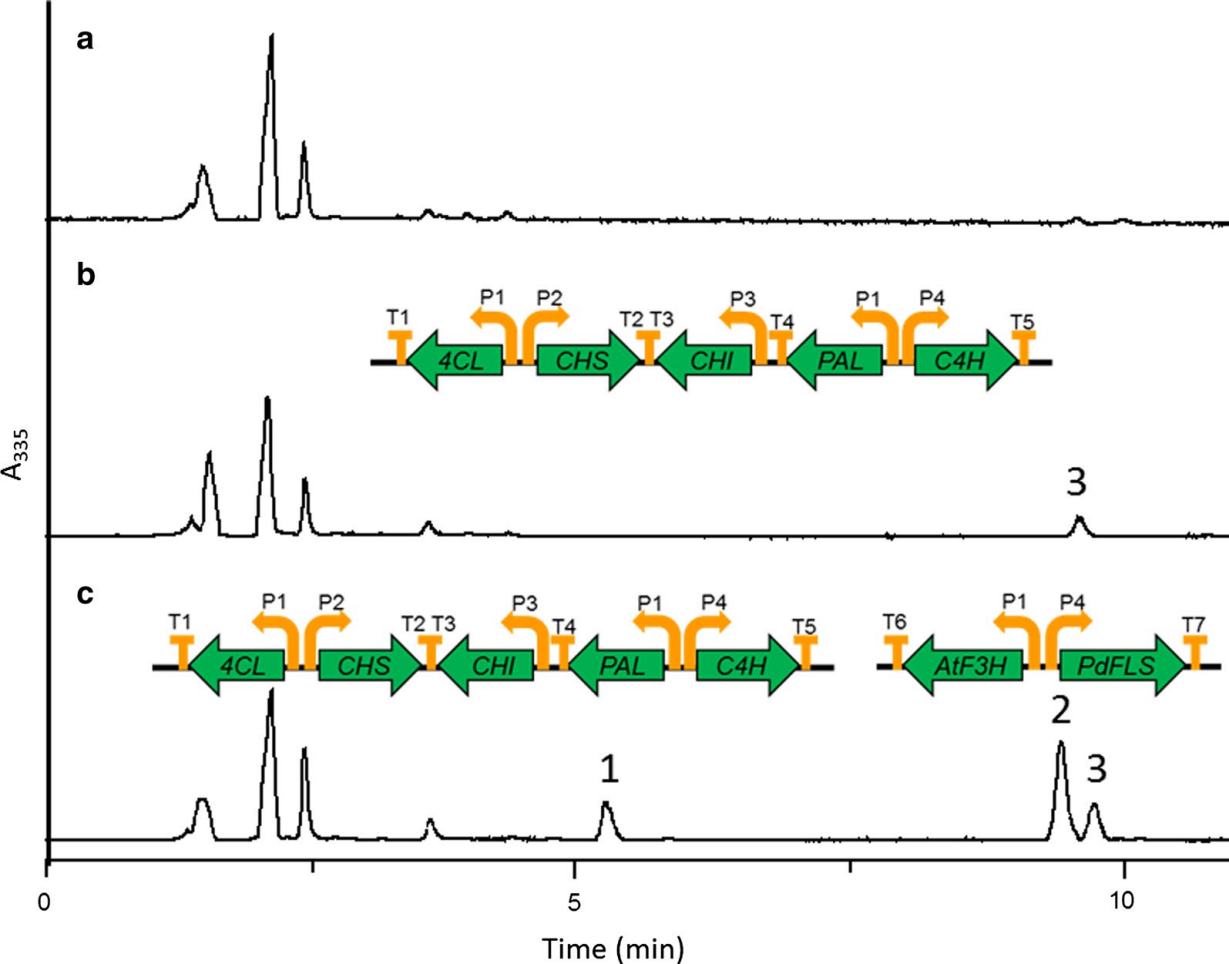
Pathway construction for *de novo* biosynthesis of kaempferol in yeast. HPLC analysis of the broth fermented by W303-1A (**a**), W3NP (**b**) and W3NP-FF (**c**) from glucose. W303-1A, wild type; W3NP, a recombinant harboring the pathway of naringenin biosynthesis; W3NP-FF, a recombinant harboring the pathway of kaempferol biosynthesis. Peak 1, dihydrokaempferol; peak 2, kaempferol; peak 3, naringenin. Organization of expressed gene clusters are shown at the top of the chromatogram. P1, *ADH1* promoter; P2, *HXT7* promoter; P3, *PGI1* promoter; P4, *TDH3* promoter; T1, *TPI1* terminator; T2, *TPG1* terminator; T3, *ADH1* terminator; T4, *FBA1* terminator; T5 *PDC1* terminator; T6, *RPS2* terminator; T7, *TDH1* terminator

Based on the high efficient kaempferol conversion with *AtF3H* and *PdFLS*, we further attempted to build a pathway for de novo biosynthesis of kaempferol. A naringenin synthesis strain, W3NP, was constructed as parent strain through expressing phenylalanine ammonia lyase, cinnamic acid 4-hydroxylase, 4-coumaric acid ligase, chalcone synthase, and chalcone isomerase from *Erigeron breviscapus* (encoded by *PAL*, *C4H*, *4CL*, *CHS*, *CHI*, respectively). Indeed, W3NP was able to synthesize naringenin from glucose, which was confirmed by LC–MS and ultraviolet (UV) absorption (Fig. 2b and Additional file 1: Figure S2). Thus, by introducing *AtF3H* and *PdFLS* to W3NP, a yeast recombinant W3NP-FF was generated, which carries a pathway for de novo kaempferol biosynthesis (Fig. 2c). In batch fermentation, W3NP produced 2.29 mg/L naringenin, while W3NP-FF produced 6.97 mg/L kaempferol, which was accompanied with 3.55 mg/L dihydrokaempferol and 3.32 mg/L naringenin (Additional file 1: Table S1). The total flavonoids production was largely improved by introducing kaempferol biosynthetic pathway (Additional file 1: Table S1). This is probably because the expression of *PdFLS* and *AtF3H* strengthened the driving force toward flavonoid synthesis from glucose. Besides, the higher kaempferol production compared to dihydrokaempferol in W3NP-FF also indicated that *PdFLS* is efficient in kaempferol production.

### Metabolic engineering for kaempferol production

**Fig. 3:**
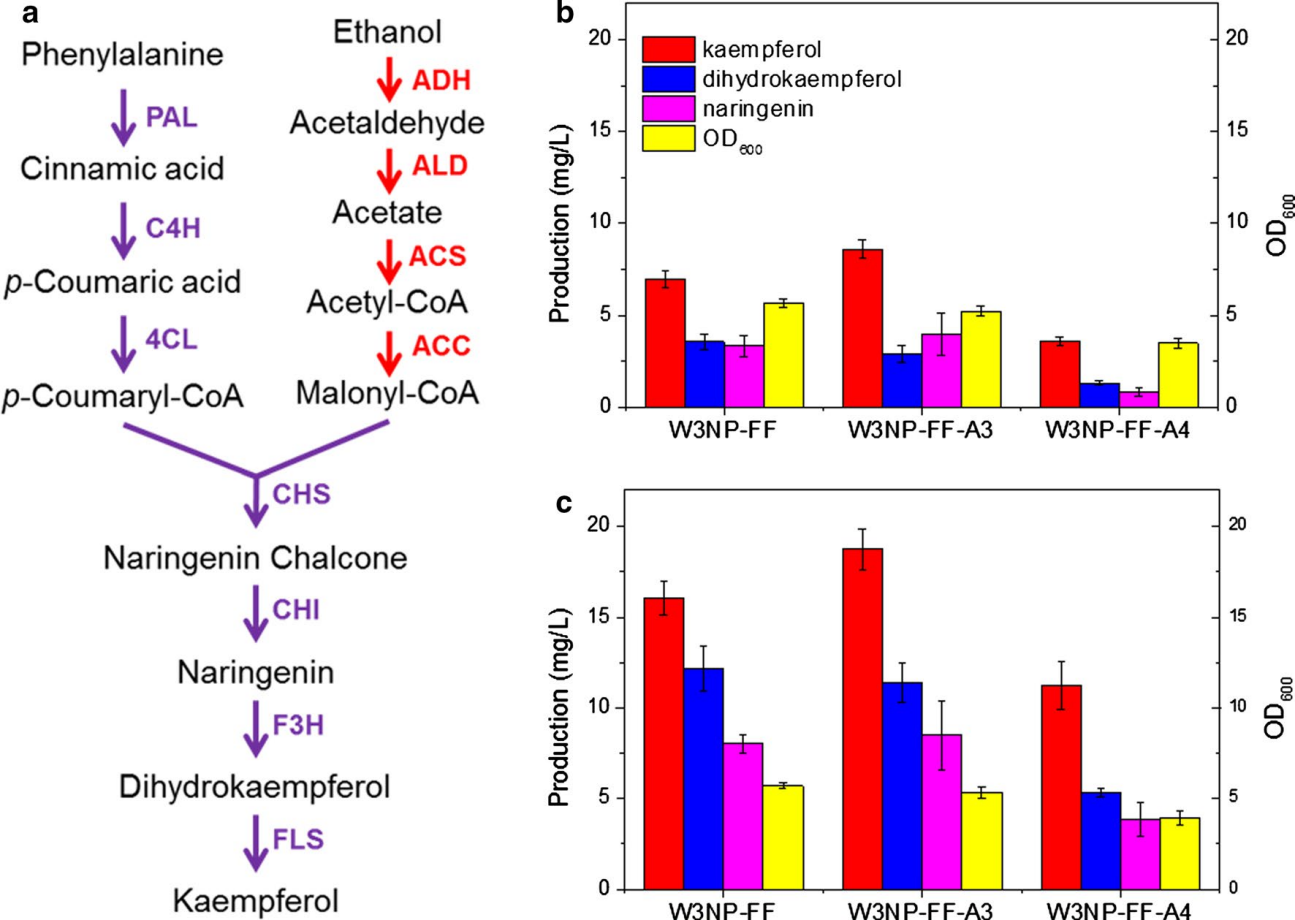
Metabolic engineering for high kaempferol production in *S. cerevisiae*. **a** Metabolic pathway and engineering scheme for kaempferol production. Purple arrows represent reactions catalyzed by exogenous enzymes from plant in kaempferol synthesis pathway. Red arrows represent reactions catalyzed by endogenous enzymes in yeast, which are involved in the precursor synthesis, and overexpressed for kaempferol production. PAL, phenylalanine ammonia lyase; C4H, cinnamic acid 4-hydroxylase; 4CL, 4-coumaric acid ligase; CHS, chalcone synthase; CHI, chalcone isomerase; F3H, flavanone 3β-hydroxylase; FLS, flavonol synthase; ADH, alcohol dehydrogenase; ALD, aldehyde dehydrogenase; ACS, acetyl-coA synthetase; ACC, acetyl-CoA carboxylase. **b** OD_600_ and production of naringenin, dihydrokaempferol and kaempferol in batch fermentation at 60 h. Glucose (20 g/L) was used as the sole carbon source. **c** OD_600_ and production of naringenin, dihydrokaempferol and kaempferol in batch fermentation at 60 h. The media were supplemented with 20 g/L glucose and 1 mM *p*-coumarate. Data are shown as average ± standard division in triplicate tests

In order to further improve the production of kaempferol, we proposed to enhance the metabolic pathway of precursors. Increasing the intracellular acetyl-CoA pool and (or) malonyl-CoA pool was beneficial for high-level flavonoids production [14, 25, 26]. The biosynthetic pathway of malonyl-CoA from ethanol consists of four steps, catalyzed by alcohol dehydrogenase, aldehyde dehydrogenase, acetyl-coA synthetase and acetyl-CoA carboxylase (encoded by *ADH2*, *ALD6*, *ACS* ^*SE*^ and *ACC1*, respectively Fig. 3a). To increase endogenous supply of acetyl-CoA or malonyl-CoA, biosynthetic genes were overexpressed in the kaempferol producing strain W3NP-FF. The gene cluster harboring *ADH2*, *ALD6*, and *ACS* ^*SE*^ expression cassettes (Additional file 1: Figure S3B), and another harboring *ADH2*, *ALD6*, *ACS* ^*SE*^ and *ACC1* (Additional file 1: Figure S3C) expression cassettes were separately transformed to W3NP-FF, generating W3NP-FF-A3 and W3NP-FF-A4, respectively. In batch fermentation, W3NP-FF-A3 produced 8.6 mg/L kaempferol from glucose, which increased 23% compared to W3NP-FF (Fig. 3b and Additional file 1: Table S1). However, W3NP-FF-A4 significantly decreased the production of kaempferol by 48%, and also decreased the production of naringenin and dihydrokaempferol by 75 and 63% respectively, compared to W3NP-FF (Fig. 3b and Additional file 1: Table S1). Besides, the *ACC1* overexpression caused a 33% reduction in final OD_600_, and reduce by 37% in specific kaempferol production compared to these of W3NP-FF-A3 in fermentation from glucose (Fig. 3b and Additional file 1: Table S1).

*ACC1* encodes cytosolic acetyl-CoA carboxylase (ACCase), catalyzing the malonyl-CoA formation from acetyl-CoA in *S. cerevisiae*, which is regarded as the rate limiting enzyme in fatty acid synthesis, and is thus tightly regulated in transcription and post-translation levels [27, 28]. In most case, overexpressing a native or a mutant *ACC1* increased the malonyl-CoA pool or the derived products [29–32]. By contrast, some studied suggested that the ACCase overexpression impaired cell growth [29, 32, 33], or did not promote the production of malonyl-CoA derived products significantly [34, 35]. The reasons for this phenomenon have generally attributed to an imbalanced synthesis of long-chain fatty acids, depletion of intermediates or high metabolic burden [27, 29, 32]. In this study, an *ACC1* mutant with Ser659Ala and Ser1157Ala, which improves ACCase activity by abolishing Snf1-dependent regulation, was driven by a *PGK1* promoter. Thus, expression of ACCase had been enhanced in both transcription and post-translation levels. The excessively increased ACCase activity may cause imbalanced synthesis of long-chain fatty acids and/or abnormal intracellular levels of acetyl-CoA and AMP, and interfere cell growth and metabolism, including flavonoids synthesis (Fig. 3b and Additional file 1: Table S1). Our results suggest that a simple and straightforward ACCase overexpression could not increase malonyl-CoA derived products as expected, and introducing a dynamic ACCase regulation or heterologous malonyl-CoA synthesis pathway would be a more promising strategy [36, 37].

Another important precursor of flavonoids biosynthesis is phenylalanine. The intracellular l-phenylalanine synthesis is tightly regulated in *S. cerevisiae* [10, 38]. Here, we supplemented *p*-coumarate in the fermentation medium in order to partially alleviate the limitation of precursor supply [10, 38]. The *p*-coumarate supplement significantly increased total flavonoids production by about 2–3.5 times for W3NP-FF, W3NP-FF-A3 and W3NP-FF-A4 (Fig. 3b, c and Additional file 1: Table S1). Particularly, the kaempferol production of W3NP-FF-A3 reached 18.76 mg/L, which is close to the highest reported value (22.57 mg/L) [39].

### Fermentation optimization for kaempferol production

**Fig. 4:**
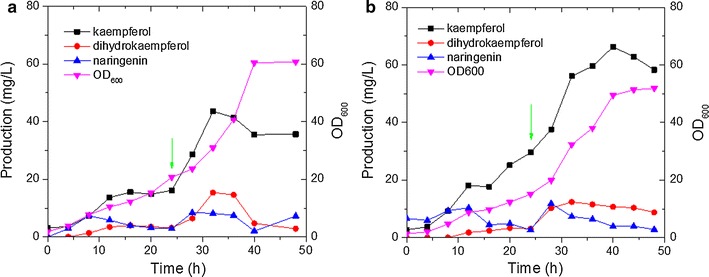
Fed-batch fermentation of kaempferol in well controlled fermenters. OD_600_ and production of naringenin, dihydrokaempferol and kaempferol are shown in fed-batch fermentation with W3NP-FF (**a**) and W3NP-FF-A3 (**b**). The fermentations were performed at 30 °C, and pH was maintained at 5.0. The feed was started when residual ethanol and glucose were completely depleted. A Quasi exponent feed strategy was adopted. Green arrow indicates 1 mM *p*-coumarate was supplemented at 24 h

Fermentation conditions, such as pH, media components, temperature, stirring speed and ventilation rate, also largely affect the titer of products in microbial cell factory. In this research, a Quasi exponent feed strategy [40] was adopted in fed-batch fermentation for high kaempferol production with W3NP-FF and W3NP-FF-A3. The OD_600_ and the production of flavonoids were measured and shown in Fig. 4a, b. Both of W3NP-FF and W3NP-FF-A3 produced much higher kaempferol in fed-batch fermentation than that in batch fermentation (Figs. 3b, c, and 4a, b). To increase substrate supply, 1 mM *p*-coumarate was supplemented after 24 h fermentation. Then the production of kaempferol, dihydrokaempferol and naringenin were consequently increased for both W3NP-FF-A3 and W3NP-FF (Fig. 4a, b). For W3NP-FF-A3, the kaempferol production continuously increased in the early 40 h (Fig. 4b); while for W3NP-FF, the kaempferol production reached the top at 32 h, but with a stationary phase from 12 to 24 h (Fig. 4a). This result showed that overexpression of acetyl-CoA synthetic pathway (*ADH2*, *ALD6*, and *ACS* ^*SE*^) not only increased the titer of kaempferol, but also improved the persistence of kaempferol production. The highest kaempferol titer in fed-batch fermentation achieved 66.29 mg/L by W3NP-FF-A3, which is 3.5-fold over that in batch fermentation (Fig. 4b, Additional file 1: Table S1 and S2). To our knowledge, it is about 2.5 times of the reported highest titer (26.57 mg/L kaempferol produced by an engineered *S. cerevisiae* [39]).

In the flavonoid synthetic pathway, malonyl-CoA and *p*-coumaroyl-CoA are two direct precursors in chalcone forming. In this research, the intracellular supply of malonyl-CoA has been enhanced by over-expression the acetyl-CoA synthetic pathway; on the other hand, the supply of *p*-coumaroyl-CoA has been satisfied by supplementing *p*-coumarate in the medium, which trends to add extra cost in fermentation. Alternatively, it has been reported that engineering the aromatic amino acid biosynthesis pathway and overexpressing the tyrosine ammonia-lyase (TAL) increased the formation and accumulation of *p*-coumaric acid in yeast cells [41]. This is an attractive strategy for promoting the production of *p*-coumaric acid derived products from glucose, and saving *p*-coumaric acid supplement in fermentation. Recently, Rodriguez et al. expressed *4CL*, *CHS*, *CHI*, *CHR* (encoding chalcone reductase), *F3H* (from *Astragalus mongholicus*), *FLS* (from *Arabidopsis thaliana*) and *CPR* (encoding cytochrome P450 reductase) and *FMO* (encoding a cytochrome P450 flavonoid monooxygenases) genes in *p*-coumaric acid over-accumulated strains, to produce several flavonoids including kaempferol [39]. Taking advantage of the enhanced *p*-coumaric acid synthesis, the engineered strain produced 26.57 mg/L kaempferol [39], which is the highest reported titer to our best knowledge. In this research, we comprehensively applied pathway construction, enzyme selection, metabolic engineering, intermediate supplement and fermentation optimization to realize and upgrade kaempferol production gradually, and achieved the highest kaempferol production at 66.29 mg/L. Here, we emphasize the combination of multi biological solutions for efficient product synthesis in a microbial cell factory.

## Conclusions

In this study, we successfully synthesized kaempferol through *S. cerevisiae* recombinants expressing FLS from *P. deltoides*, which resulted in higher kaempferol production than those from *A. thaliana*, *C. unshiu*, *M. domestica*, and *Z. mays*. Through expressing *PAL*, *C4H*, *4CL*, *CHS*, *CHI*, *AtF3H* and *PdFLS*, we constructed a yeast recombinant that synthesizes kaempferol de novo. Furthermore, we demonstrated that overexpressing the acetyl-CoA synthetic pathway (consisted of *ADH2*, *ALD6* and *ACS* ^*SE*^) in cytoplasm and *p*-coumarate supplement in media would significantly increase kaempferol titer of the recombinant. Fermentation conditions are also closely related to the kaempferol biosynthesis that fed-batch fermentation in a bioreactor with a Quasi exponent feed strategy largely improved kaempferol production compared to batch fermentation in flask. The titer of kaempferol reached up to 66.29 mg/L after 40 h fermentation. To our knowledge, it is the highest kaempferol titer in microbial cell factories currently.

## Methods

### Genes and strains

Flavonol synthases from *A. thaliana* (accessing number NP_196481.1, encoded by *AtFLS*), *C. unshiu* (accessing number: Q9ZWQ9.1, encoded by *CitFLS*), *M. domestica* (accessing number: NP_001306179.1, encoded by *MdFLS*),*Z. Mays* (accessing number: XP_008646309.1, encoded by *ZmFLS*) and *P. deltoides* (TIGR accession number: TC74233 [23], encoded by *PdFLS*) were investigated in this work. Flavanone 3β-hydroxylase (F3H) was from *A. thaliana* (encoded by *AtF3H*, accessing number: NP_190692.1). *PAL*, *C4H*, *4CL*, *CHS* and *CHI* were from *Erigeron breviscapus* (Vant.) Hand-Mazz. (kindly provided by Guang-Hui Zhang [42]). The codon usage of *CitFLS*, *MdFLS*, *ZmFLS*, *PdFLS*, *PAL*, *C4H*, *4CL*, *CHS* and *CHI* were optimized for *S. cerevisiae* (Additional file 1: Table S3) and synthesized by a local company.

*Escherichia coli* DH5α was used for gene cloning. *S. cerevisiae* W303-1A was used for genetic engineering and kaempferol production. All strains and plasmids used in this study are shown in Table 1.

**Table 1:** Plasmids and strains used in this study

Plasmid or strain	Genotype	Source or reference
Plasmids		
YCplac22	*Amp Trp1*	Gietz [50]
YCplac33	*AmpURA3*	Gietz [50]
Y22-T4	T_*ADH1*_/T_*TDH3*_	This work
Y22-AtF3H	P_*PGK1*_-*AtF3H*-T_*ADH1*_	This work
Y22-T4-AtF3H-AtFLS	P_*PGK1*_-*AtF3H*-T_*ADH1*_/P_*TDH3*_-*AtFLS*-T_*TDH1*_	This work
Y22-T4-AtF3H-CitFLS	P_*PGK1*_-*AtF3H*-T_*ADH1*_/P_*TDH3*_-*CitFLS*-T_*TDH1*_	This work
Y22-T4-AtF3H-MdFLS	P_*PGK1*_-*AtF3H*-T_*ADH1*_/P_*TDH3*_-*MdFLS*-T_*TDH1*_	This work
Y22-T4-AtF3H-PdFLS	P_*PGK1*_-*AtF3H*-T_*ADH1*_/P_*TDH3*_-*PdFLS*-T_*TDH1*_	This work
Y22-T4-AtF3H-ZmFLS	P_*PGK1*_-*AtF3H*-T_*ADH1*_/P_*TDH3*_-*ZmFLS*-T_*TDH1*_	This work
Y33-ALAC-ADH2	P_*TEF2*_-*ACS* ^*SE*^-T_*RPS2*_/P_*PGK1*_-*ALD6*-T_*TDH1*_/P_*HXT7*_-*ADH2*-T_*RPL9A*_	This work
Y33-ALAC-ACAD	P_*TEF2*_-*ACS* ^*SE*^-T_*RPS2*_/P_*PGK1*_-*ALD6*-T_*TDH1*_/P_*HXT7*_-A*DH2*-T_*RPL9A*_/P_*PGK1*_-*ACC1*-T_*CCW12*_	This work
Strains		
W303-1A	*MAT*a *leu2*-*3112 ura3*-*1 trp1*-*92 his3*-*11,15 ade2*-*1 can1*-*100*	Thomas [51]
W3-AtF3H	W303-1A carrying Y22-AtF3H	This work
W3-AtFLS	W303-1A carrying Y22-T4-AtF3H-AtFLS	This work
W3-CitFLS	W303-1A carrying Y22-T4-AtF3H-CitFLS	This work
W3-MdFLS	W303-1A carrying Y22-T4-AtF3H-MdFLS	This work
W3-PdFLS	W303-1A carrying Y22-T4-AtF3H-PdFLS	This work
W3-ZmFLS	W303-1A carrying Y22-T4-AtF3H-ZmFLS	This work
W3NP	*YORWdelta17*::*His3*/P_*ADH1*_-*4CL*-T_*TPI1*_/P_*HXT7*_-*CHS*-T_*TPG1*_/P_*PGI1*_-*CHI*-T_*ADH1*_/P_*ADH1*_-*PAL*-T_*FBA1*_/P_*TDH3*_-*C4H*-T_*PDC1*_	This work
W3NP-FF	W3NP carrying Y22-T4-AtF3H-PdFLS and YCplac33	This work
W3NP-FF-A3	W3NP carrying Y22-T4-AtF3H-PdFLS and Y33-ALAC-ADH2	This work
W3NP-FF-A4	W3NP carrying Y22-T4-AtF3H-PdFLS and Y33-ALAC-ACAD	This work

### Gene cloning and plasmid construction

All DNA manipulations were performed according to standard procedures [43]. Phusion High-Fidelity DNA Polymerase (Thermo Fisher Scientific) was used for PCR amplification. A DNA fragment, named Ter-4, was designed for Golden gate cloning, which contained two terminators (*T*_*RSP2*_ and *T*_*TDH1*_), being separated by two *Bsa*I digest sites (the scheme was shown in Additional file 1: Figure S3A). The Ter-4 fragment was synthesized by a local company (Synbio Technologies), and cloned to pUC57. Then the Ter-4 fragment and YCplac22 were amplified (primers: T4-YCP-F/T4-YCP-R, Y22-T4-F/Y22-T4-R, Additional file 1: Table S4) and assembled together using CPEC (Circular Polymerase Extension Cloning [44]), generating Y22-T4 (Additional file 1: Figure S4), the backbone for FLS and F3H expression. In CPEC, the vector and the insert share overlapping regions at the ends, and the hybridized insert and vector extend using each other as a template until they complete a full circle in a PCR system, and finally the PCR product is transformed directly to DH5α. For assembly of gene expression cassettes, 3 DNA fragments, including a head-to-head promoter fragment, *AtF3H* and each of the *FLS* genes (*AtFLS*, *CitFLS*, *MdFLS*, *PdFLS*, *ZmFLS*), were amplified (primers: P1-F/P1-R for promoter fragment, AtF3H-GG2-F/AtF3H-GG2-R for *AtF3H*, FLS-GG1-F/FLS-GG1-R for each *FLS* gene, Additional file 1: Table S4) respectively, and ligated to Y22-T4 through golden gate cloning [45], generating FLS and F3H expression cassettes (Additional file 1: Figures S3A, S4). Thus, Y22-T4-F3H-AtFLS, Y22-T4-AtF3H-CitFLS, Y22-T4-AtF3H-MdFLS, Y22-T4-AtF3H-PdFLS and Y22-T4-AtF3H-ZmFLS was constructed. Then, expression cassette of *AtF3H* was amplified (primer: F3H-CPF/F3H-CPR, Additional file 1: Table S4) and ligated to YCplac22 through CPEC [44], resulting in Y22-AtF3H. Similarly, expression cassettes of *ALD6*, *ACS* ^*SE*^ [46], *ADH2* and *ACC1* (a mutant with Ser659Ala and Ser1157Ala, [29]) were constructed through golden gate cloning. Then, the cassettes of *ALD6*, *ACS* ^*SE*^ and *ADH2* were inserted into YCplac33 through Gibson assembling [47] (primers: Y33-F/Y33-R, ALAC-GibF/ALAC-GibR, ADH2-GibF/ADH2-GibR, Additional file 1: Table S4), resulting in the plasmid Y33-ALAC-ADH2 (Additional file 1: Figure S3B). Cassettes of *ALD6*, *ACS* ^*SE*^, *ADH2* and *ACC1* were inserted into YCplac33 through Gibson assembling [47] (primers: Y33-F/Y33-R, ALAC-GibF/ALAC-GibR, ACAD-GibF/ADH2-GibR, Additional file 1: Table S4), generating the plasmid Y33-ALAC-ACAD (Additional file 1: Figure S3C).

### Strain construction

To generate a naringenin-producing *S. cerevisiae* recombinant, a modularized two-step (M2S) chromosome integration technique [45] was applied for *PAL*, *C4H*, *4CL*, *CHS* and *CHI* expression. In brief, *PAL* and *C4H*; *4CL* and *CHS*; and *CHI* was ligated with promoters and terminators respectively through golden gate cloning, forming the expression cassettes. Then, DNA fragments harboring these cassettes were transformed together with His marker and homologous arms into wild strain (W303-1A), and integrated into genome through DNA assembler [48] (Additional file 1: Figure S3D), resulting in the naringenin-producing recombinant, W3NP. Then, Y22-T4-AtF3H-PdFLS was co-transformed into W3NP with YCplac33, Y33-ALAC-ADH2 and Y33-ALAC-ACAD respectively, resulting in W3NP-FF, W3NP-FF-A3 and W3NP-FF-A4.

### Media and culture condition

*Escherichia coli* was grown in Luria–Bertani (LB) medium at 37 °C. Ampicillin (50 μg/mL) was added to the medium when required. Yeast strains were grown at 30 °C in YPD medium (10 g/L yeast extract, 20 g/L Bacto peptone, and 20 g/L glucose) or defined mineral medium (YSCD), containing 6.7 g/L yeast nitrogen base (YNB) without amino acids (Difco, Detroit, Michigan), supplemented with the appropriate auxotrophic requirements and 20 g/L glucose. YSCD supplemented with 5 mM sodium ascorbate and 0.1 mM Fe_2_SO_4_ was used for batch fermentation in flask, and 1 mM *p*-coumarate was added when required.

The medium for fed-batch fermentation contained: 20 g/L glucose, 15 g/L (NH_4_)_2_SO_4_, 8 g/L KH_2_PO_4_, 6.2 g/L MgSO_4_·7H_2_O, 12 mL/L vitamin solution and 10 mL/L trace metal solution, where the vitamin solution contained 0.05 g/L biotin, 1 g/L calcium pantothenate, 1 g/L nicotinic acid, 25 g/L inositol, 1 g/L thiamine HCl, 1 g/L pyridoxal HCl, 0.2 g/L *p*-aminobenzoic acid and 2.5 g/L adenine; the trace metal solution contained: 5.75 g/L ZnSO_4_·7H_2_O, 0.32 g/L MnCl_2_·4H_2_O, 0.47 g/L CoCl_2_·6H_2_O, 0.48 g/L Na_2_MoO_4_·2H_2_O, 2.9 g/L CaCl_2_·2H_2_O, 2.8 g/L FeSO_4_·7H_2_O and 80 mL/L 0.5 M EDTA, pH 8.0. The same medium was used for seed culture in fed-batch fermentation [49].

### Fermentation

#### Whole cell catalysis

Inoculum was cultured in YSCD medium at 30 °C for 12 h. The pre-culture was refreshed in 20 mL YSCD to OD_600_ = 1. Cells were then collected and re-suspended in 3 mL YSCD supplemented with 5 mM sodium ascorbate, 0.1 mM Fe_2_SO_4_ and 100 mg/L (*2S*)-naringenin (Solarbio Life Sciences), to a final cell concentration of OD_600_ = 5. The reaction was incubated at 30 °C in an orbital shaker (220 rpm) for 24 h.

#### Batch fermentation

Inoculum was cultured in YSCD medium at 30 °C for 12 h. The pre-culture was then used to inoculate 20 mL batch fermentation medium (YSCD supplemented with 5 mM sodium ascorbate and 0.1 mM Fe_2_SO_4_) in 250 mL shaker flasks to a final cell concentration of OD_600_ = 1. One mM *p*-coumarate was added as precursor when required. The fermentation lasted for 60 h.

#### Fed-batch fermentation

Glycerol-stocked cells were inoculated into 40 mL YSCD and cultured at 30 °C, 220 rpm for 24 h. The culture was transferred to 360 mL fed-batch fermentation medium, and cultured for another 24 h. 300 mL seeds were inoculated to 1.5 L fed-batch fermentation medium in Baoxin bioreactor with a maximal working volume of 3 L. The fermentations were performed at 30 °C, and pH was maintained at 5.0 with automatic addition of ammonium hydroxide or 1 M H_2_SO_4_. The agitation rate was kept between 300 and 800 rpm, and the air flow was set as 1.5 vvm. The dissolved oxygen concentration was controlled above 40% throughout regulation the agitation rate [40]. The feed was started after residual ethanol and glucose were completely depleted. A Quasi exponent feed strategy was adopted as described [40]. Feed reagent contained: 386 g/L glucose, 9 g/L KH_2_PO_4_, 5.12 g/L MgSO_4_·7H2O, 3.5 g/L K_2_SO_4_, 0.28 g/L Na_2_SO_4_, 5 g/L adenine, 12 mL/L vitamin solution and 10 mL/L trace metal solution. 1 mM *p*-coumarate was supplemented at 24 h.

### Detection and quantification of the products

Flavonoids were extracted directly from the culture with an equal volume of methanol. The extraction were analyzed by High Performance Liquid Chromatography (HPLC, Agilent), using Phenomenex Kinetex Biphenyl Column (5 μm, 250 × 4.6 mm) equipped with a photodiode array detector. The mobile phase consisted of acetonitrile and water (0.1% phosphoric acid) using a gradient elution of 30–40% acetonitrile for 10 min, 40–95% acetonitrile for 2.5 min,95–30% acetonitrile for 2.5 min and 30% acetonitrile for 5 min, at flow rate of 1 mL/min. Samples were analyzed by LC–MS using a Thermo U3000-LTQ XL (Thermo Scientific) system coupled to the ion trap mass spectrometer with an ESI source operating in the positive mode. LC–MS analysis was operated with the same LC operation method, except 0.1% formic acid was substitute to phosphoric acid. Quantification of the kaempferol was based on the peak areas of absorbance at 335 nm. Quantification of dihydrokaempferol and naringenin was based on the peak areas of absorbance at 290 nm.

## Additional file

**Additional file 1: Table S1.** The production of flavonoids from engineered yeasts in batch fermentation. **Table S2.** Maximum OD_600_ and production of kaempferol, dihydrokaempferol and naringenin of W3NP-FF and W3NP-FF-A3 in fed-batch fermentation. **Table S3.** Optimized gene sequence for *S. cerevisiae.* **Table S4.** Primers used in this work. **Figure S1.** Mole ratio of kaempferol to dihydrokaempferol (A) and mole ratio of kaempferol to total naringenin (B) in whole cell catalysis with the recombinants expressing FLS orthologs. **Figure S2.** Identification of naringenin produced by W3NP from glucose. A, LC-MS analysis of naringenin produced by W3NP. B, UV absorption of naringenin produced by W3NP (solid line) and standard (dash line). **Figure S3.** Scheme of plasmids construction and gene manipulation for kaempferol production. A, Sketch drawing for F3H and FLS expression. B, Y33-ALAC-ADH2, a plasmid expressing *ALD6*, *ACS* ^*SE*^ and *ADH2*. C, Y33-ALAC-ACAD, a plasmid expressing *ALD6*, *ACS* ^*SE*^, *ADH2* and *ACC1*. D, Genome manipulation in W3NP for naringenin production. **Figure S4.** Scheme of cassette assembly through golden gate cloning. A. The design of Ter-4 fragment and the cloning vector Y22-T4. The vector Y22-T4, DNA fragments of promoter, F3H and FLS were digested by *Bsa*I and ligated through sticky ends.
