# Supplementary material for: Biosynthesis and engineering of kaempferol in Saccharomyces cerevisiae
Source: Microb Cell Fact. 2017 Sep 26;16:165. doi: 10.1186/s12934-017-0774-x (PMC5615808; doi:10.1186/s12934-017-0774-x)
Supplement: Supplementary file 1 — Additional file 1: Table S1. The production of flavonoids from engineered yeasts in batch fermentation. Table S2. Maximum OD600 and production of kaempferol, dihydrokaempferol and naringenin of W3NP-FF and W3NP-FF-A3 in fed-batch fermentation. Table S3. Optimized gene sequence for S. cerevisiae. Table S4. Primers used in this work. Figure S1. Mole ratio of kaempferol to dihydrokaempferol (A) and mole ratio of kaempferol to total naringenin (B) in whole cell catalysis with the recombinants expressing FLS orthologs. Figure S2. Identification of naringenin produced by W3NP from glucose. A, LC-MS analysis of naringenin produced by W3NP. B, UV absorption of naringenin produced by W3NP (solid line) and standard (dash line). Figure S3. Scheme of plasmids construction and gene manipulation for kaempferol production. A, Sketch drawing for F3H and FLS expression. B, Y33-ALAC-ADH2, a plasmid expressing ALD6, ACS SE and ADH2. C, Y33-ALAC-ACAD, a plasmid expressing ALD6, ACS SE, ADH2 and ACC1. D, Genome manipulation in W3NP for naringenin production. Figure S4. Scheme of cassette assembly through golden gate cloning. A. The design of Ter-4 fragment and the cloning vector Y22-T4. The vector Y22-T4, DNA fragments of promoter, F3H and FLS were digested by BsaI and ligated through sticky ends. [file 12934_2017_774_MOESM1_ESM.docx]

Supplementary material

**Table S1 The production of flavonoids from engineered yeasts in batch fermentation.**

| **Strain** | **OD_600_** | **Kaempferol mg/L** | **Dihydro -kaempferol mg/L** | **Naringenin mg/L** | **Total flavonoids^a^ mg/L** | **Specific kaempferol production^b^**  **mg/g DCW** |
| --- | --- | --- | --- | --- | --- | --- |
| **Fermentation from glucose** | | | | | | |
| W3NP | 6.81±0.47^a^ | N.D. | N.D. | 2.29±0.31^b^ | 2.29 |  |
| W3NP-FF | 5.65±0.21^b^ | 6.97±0.44^b^ | 3.55±0.41^a^ | 3.32±0.56^a^ | 13.84 | 4.36 |
| W3NP-FF-A3 | 5.23±0.28^b^ | 8.60±0.51^a^ | 2.89±0.47^a^ | 3.98±1.17^a^ | 15.47 | 5.81 |
| W3NP-FF-A4 | 3.48±0.27^c^ | 3.61±0.22^c^ | 1.30±0.12^b^ | 0.82±0.21^c^ | 5.73 | 3.66 |
| **Fermentation from glucose and *p*-coumarate** | | | | | | |
| W3NP-FF | 5.71±0.15^a^ | 16.05±0.92^b^ | 12.15±1.25^a^ | 8.03±0.49^a^ | 36.23 | 9.93 |
| W3NP-FF-A3 | 5.34±0.28^a^ | 18.76±1.12^a^ | 11.38±1.07^a^ | 8.51±1.90^a^ | 38.65 | 12.41 |
| W3NP-FF-A4 | 3.92±0.39^b^ | 11.23±1.29^c^ | 5.32±0.22^b^ | 3.85±0.93^b^ | 20.40 | 10.13 |

The data were obtained at 60 hours in fermentation. YSCD media was used, and 1 mM *p*-coumarate was supplemented when required. Data are shown as average ± standard division in triplicate tests. Different superscript letter indicates significant difference in Tukey analysis (α=0.05). N.D., not detected.

^a^Total flavonoids is defined as the sum of productions of kaempferol, dihydrokaempferol and naringenin.

^b^A biomass conversion ratio of 1 OD=0.283 g/L Dry Cell Weight (DCW) was applied in Specific kaempferol production calculation.

**Table S2. Maximum OD_600_ and production of kaempferol, dihydrokaempferol and naringenin of W3NP-FF and W3NP-FF-A3 in fed-batch fermentation.**

| **Strain** | **OD_600_** | **Kaempferol mg/L** | **Dihydrokaempferol mg/L** | **Naringenin mg/L** |
| --- | --- | --- | --- | --- |
| W3NP-FF | 60.70 | 43.58 | 15.39 | 8.43 |
| W3NP-FF-A3 | 51.85 | 66.29 | 12.39 | 11.83 |

**Table S3. Optimized gene sequence for *S. cerevisiae***

| **Name** | **Optimized sequence** |
| --- | --- |
| *CitFLS* | ATGGAAGTTGAACGCGTTCAAGCAATTGCGAGCCTGAGTCATAGTAACGGTACCATTCCGGCAGAGTTCATTCGTCCGGAAAAAGAACAGCCGGCAAGTACCACCTATCACGGTCCGGCACCGGAAATTCCGACCATTGATCTGGACGATCCGGTTCAGGATCGCCTGGTTCGTAGTATTGCAGAAGCGAGCCGCGAGTGGGGTATCTTTCAGGTTACCAACCACGGTATTCCGAGCGATCTGATCTGCAAACTGCAGGCGGTTGGCAAAGAATTTTTCGAACTGCCGCAGGAAGAAAAAGAAGTTTATAGCCGTCCGGCTGACGCAAAAGATGTTCAGGGTTACGGCACCAAACTGCAGAAAGAAGTCGAGGGCAAAAAATCCTGGGTCGATCACCTGTTTCATCGCGTTTGGCCGCCGAGTAGCATTAACTACCGCTTTTGGCCGAAAAACCCGCCGAGTTATCGCGCAGTTAACGAAGAGTACGCCAAATACATGCGCGAAGTCGTGGATAAACTGTTCACCTACCTGTCTCTGGGTCTGGGTGTAGAAGGCGGCGTTCTGAAAGAAGCAGCAGGCGGCGACGATATCGAATATATGCTGAAAATCAACTACTATCCGCCGTGTCCGCGTCCGGATCTGGCACTGGGGGTTGTTGCACATACCGATCTGTCTGCACTGACCGTTCTGGTTCCGAACGAAGTTCCGGGTCTGCAGGTTTTTAAAGACGACCGCTGGATCGACGCGAAATATATTCCGAACGCCCTGGTTATCCATATTGGCGATCAGATCGAGATCCTGAGCAACGGCAAATACAAAGCGGTTCTGCATCGTACCACCGTTAACAAAGACAAAACCCGCATGAGTTGGCCGGTTTTTCTGGAACCGCCGGCAGATACCGTTGTAGGTCCGCTGCCGCAACTGGTTGACGACGAAAACCCGCCGAAATACAAAGCGAAAAAATTCAAAGACTACAGCTACTGCAAACTGAACAAACTGCCGCAGTAA |
| *MdFLS* | ATGGGTGTTGAAAGCGTTGAACGCGAACGCGAAAGTAACGAAGGTACCATCCCGGCGGAATTTATCCGTAGCGAAAACGAACAGCCGGGTATTACCACCGTTCACGGTAAAGTCCTGGAAGTTCCGATCATTGATTTCAGCGATCCGGACGAAGAGAAACTGATCGTCCAGATTACCGAAGCGAGTTCCAACTGGGGTATGTACCAGATCGTCAACCACGATATCCCGAGCGAAGTCATTAGCAAACTGCAGGCGGTTGGCAAAGAGTTCTTCGAACTGCCGCAGGAAGAGAAAGAAGCATACGCGAAACCGCCGGATTCTGGTAGTATTGAAGGCTACGGCACCAAACTGTTCAAAGAGATCAGCGAAGGCGATACCACCAAAAAAGGCTGGGTGGATAACCTGTTCAACAAAATCTGGCCGCCGTCTGTTGTCAACTATCAGTTTTGGCCGAAAAACCCGCCGAGTTATCGCGAAGCAAACGAAGAATACGCGAAACACCTGCACAACGTCGTCGAAAAACTGTTCCGTCTGCTGTCACTGGGTCTGGGTCTGGAAGGTCAGGAACTGAAAAAAGCAGCAGGCGGCGATAATCTGGAATACCTGCTGAAAATCAACTACTACCCGCCGTGTCCGCGTCCGGATCTGGCACTGGGGGTTGTTGCTCACACCGATATGAGCACCGTTACCATTCTGGTTCCGAACGACGTTCAGGGTCTGCAGGCGTGCAAAGACGGTCGTTGGTACGACGTCAAATACATCCCGAACGCCCTGGTTATCCATATCGGCGATCAGATGGAGATCATGAGCAACGGCAAATATACCAGCGTTCTGCATCGTACCACCGTCAACAAAGACAAAACCCGCATCAGCTGGCCGGTTTTTCTGGAACCGCCGGCAGATCATGTAGTTGGTCCGCATCCGCAACTGGTTAACGCAGTCAACCAGCCGAAATACAAAACCAAAAAATACGGCGACTACGTCTACTGCAAAATCAACAAACTGCCGCAGTAA |
| *ZmFLS* | ATGGGTGGTGAAACCCATCTGAGCGTTCAAGAACTGGCAGCAAGTCTGGGCGCACTGCCGCCGGAATTTGTACGTAGCGAACAGGATCAGCCGGGTGCAACCACCTATCGCGGCGCAGCAGTTCCGGACGCACCGGTTATTGATATTAGCGAACCGGGTTTTGGCGCACGTATGGCAGCTGCTGCTCGCGAGTGGGGTCTGTTTCAAGTTGTTAATCATGGCGTTCCGTCTGCAGCAGTTGCAGAACTGCAACGCGTTGGTCGCGCATTTTTCGCGCTGCCGACCGAAGAAAAAGAACGTTACGCGATGGACCCGGCATCTGGCAAAATTGAAGGCTACGGTACCAAACTGCAACGCGATCTGGAGGGCAAAAAGACCTGGAACGACTTCTTCTTTCACGTTGTTGCGCCGCCGGAAAAAGTTGATCATGCAGTTTGGCCGCGTAGTCTGGCAGGTTATCGCGAAGCGAACGAAGAGTATTGCCGTCATATGCAACGTCTGACCCGCGAACTGTTTGAACATCTGAGTCTGGGTCTGGGTCTGCATGGCGGTGCAATGGCAGAAGCATTTGGTGGCGACGGTCTGGTATTTCTGCAGAAAATCAACTTCTACCCGCCGTGTCCGCAACCGGAACTGACCCTGGGCGTTGCTCCGCATACCGATATGAGTACCCTGACCGTTCTGGTTCCGAATGAAGTTCAGGGCCTGCAGGTTTTCAAAGACGGTCAGTGGTACGAAGCGAAATACGTTCCGGACGCACTGATTGTTCATATTGGCGACCAGATCGAGATCTTCAGCAACGGCGCATACAAAGCAGTTCTGCATCGCACCACCGTTAACAAAGAAAAAACCCGCATGTCCTGGCCGATGTTTGTAGAACCGCCGGGCGAACTGGTTGTTGGTCCGCATCCGAAACTGGTTACCGAAGAAAGCCCGGCGAAATACAAAGCGAAAAAATACAAAGACTACCAGCACTGCAAAATCAACAAACTGCCGATGTAA |
| *PdFLS* | ATGGAGTTCGACAGAGTTCAAGCCATCGCTAGCTTGTCCTTCGATAAAGAAACTATCCCAGAAGAATTTATCAGACCAGAGAAAGAACAACCAGCTGCTACTACTTTTCACGGTCCAGTTCCAGAGATCCCAACTATCGACTTGAACGACCCAAACCCAGAGAACTTGGTCAGGTTGATCGCTGACGCTTCCAAAGAGTGGGGTATTTTCCAAGTTGTCAACCACGGTATCCCATCCGATTTGATCGCAAAGTTGCAGGACGTCGGTAAGAAGTTCTTCGAGTTGCCACAAGAAGAAAAAGAAGTTTACGCTAAGCCACACGACTCCAAGTCTATCGAAGGTTACGGCTCCAAGTTGCAGAACAACCCACAAGTCAAGAAGTCCTGGGTCGATCACTTGTTCCACATCATCTGGCCACCATCTTCCATCAACTACCAGTTCTGGCCAAACAACCCACCATCCTACAGAGAAGTCAACGAAGAATATGCCAAGTACATGAGAGAGGTTACCGACAAGTTGTTCACCGCTTTGTCCTTGGGTTTGGGTTTGGAAGGTCACGCCCTTAAAGAAGGTGCTGGAGGCGAAGAAATTGAGTACATGTTGAAGATCAACTACTACCCACCTTGCCCAAGACCAGATTTGACTTTGGGCGTTGCTGCTCATACCGATTTGTCCGCCTTGACCATCTTGGTTCCAAACGAAGTCCCAGGTTTGCAGATCTTCAAGGACGGTAACTGGTTCGAAGCCAAGTACATCCCAAACGCCTTGATCATCCACATCGGCGATCAAATCGAGATCTTGTCCAACGGTAAGTACAAGGCCGTCTTGCATAGAACCACCGTTGCTAAGGACAAGGCTAGAATGTCCTGGCCAGTTTTCTTGGAACCACCAGGCGAATTGGTTGTCGGTCCATTGCCACACTTGATCAACGAAGACAACCCACCAAAGTTCAAGGCCAAGAAGTTCGAGGACTACATGTACTGCAAGTTGAACAGGTTGCCAcAGTAA |
| *PAL* | TTAACAGATTGGCAATGGAGCACCGTTCCAACCTTCAACACATTCCAACAATGGGTCGATGATTTGACCCTTACACATAGCAGTGAAAACTCTGTCGAATTCTTCACCTGGAGAAGTAACCTTTTCACCAGTCAAGTAAGAACCACCCAATTCTTCTCTAACGAATCTGTACAATGGGTAAGATCTACAAGCCTTGATTCTGTTTGGGATAGACAAAGTGTCGTTTTCGAAAGCAACTCTAACAGATTCAACTTCCTTTGGCAAGATAGCCTTCAATTCTTCTTCGAAAGTAGCGATCTTTTGGAAGATAGAAGTGTTAGTGTTCTTTTCAGTTTCACCGTTGTTCAAAGCGTGGTCAACCAAAACTTGTCTCAACTTTTGCATCAATGGGTAAGTAGCCAAACATGGGTCGTCGATGTAAGCGAAAACGTATTCTCTGTCAACAACTCTCAACAAGTCCTTTTCACAGAATCTAGATGGGTGCAATTCACCGTTAACACCCATAGTCAAAACCTTCTTAGCAACTTGAGAAACAGTGTTCTTAACAACAGACTTCATGTTTTCTTCCAAGTGTCTCAAGTCAACAGATTGACACAAAGCAACCAAGTAAGTAGAAGACATCAACTTCAAGATGTCAACAGCTTCAGCAGTCTTTCTAGCAGAGATCAAACCCAAAGAGTTAACGTCTTGGTTGTGTTGTTCAGCAGATTGAACGTGGTTAGTAACTGGGTTAGCCAAGAATTGCAATTCAGAACAGTAAGAAGCCATAGCGATTTCACCACCCTTGAAACCGTAGTCCAAAGATGGGTTTCTACCACCAGACAAGTTAGATGGCAAACCGTTGTTGTAGAAGTCGTTAACCAATTCAGAGAATTGAGCGAACATCAACTTACCGATAGCAGCGATAGCCAATCTAGTGTTGTCCATAGAAACACCGATTGGAGTACCTTGGAAGTTACCACCGTGCAAAGCCTTGTTTCTAGAAACGTCGATCAATGGGTTGTCGTTAACAGAGTTGATTTCTCTTTCGATCATCTTAGTAGAAGATCTGATAACTTCGATCAATGGACCCAACCATTGTGGAGAAGTTCTCAAAGCGTATCTGTCTTGCTTTGGCTTTTGCAATGGGTCCATTTCGTGAACCTTTTGAGCAGCCTTAACGTAGTCAGAACCGTCCAAGATGTATTCCATGATAGCAGCAGCTTCGATTTGACCTGGGTGGTGCTTCAACTTGTGAGTCAAGTGGTCAGTGAATTCTGGCTTACCTTGCATAACTTCAGCGAAGATAGCAGACAAAACTTCAGACAACAAAGCCAAAACGTTAGCTTCGAACAAAACCATAGAAGCCATACCAGAACCAACAGCAGTACCGTTAACCAAAGCCAAACCTTCCTTTGGTTGCAATTCGAAGAAACCACCTTCAACACCAGCCAACTTGAAAGCCTTTTCAGCGTTCAAAACTTCACCGTTTGGACCAACAGCCTTAGAGTTTGGTCTACCAGTCAACAAACCAGCGATGTAAGACAATGGAACCAAGTCACCAGAAGCAGTGATAGTACCTCTCAATGGCAAACATGGAGTGATGTTAGTGTTCAAGAACTTAGTGATAGCTTCCAAGATTTCGAATCTGATACCAGAGTAACCTTGCAACAAAGTGTTGATTCTAACCAACATAGCAGCTCTAGTAGCAGAGTGTGGCAAAGTGTGAGAAGATTCAGTACCGTTACCGAAGATACCAGCGTTCAAGAATCTGATCAATTCCTTTTGCAAAGCACCACCGTTCTTAGTTCTTCTGTGAGAAGTAGCACCGAAACCAGTAGTAACACCGTAAGAGTCAGTACCCTTGTTCATAGATTCCATAACCCAGTTAGAAGAAGCCTTAACACCAGCTCTAGCAGATTCAGACAATTCAACCTTAACAACAGAAGAGTCACCAGCAGCAGCGATACCAGCAACTTGAGAAACAGTCAAAGTTTCACCACCCAACTTAACCAATGGCTTTCTGAATTCAGCAACCATTCTCTTAACTTCGTCCAAGTGAGAACCAGTCAAAGCTTCAGCAGCAACACCCCAGTTCAATGGGTCCTTGTTGATACACAATTCAGAAGCAACACCGTTAGCGTGACCGTTTTCCAT |
| *C4H* | ATGGACTTGTTGTTGTTGGAAAAGACTTTGATCGCTTTGTTCGCTACTATCATCATCACTATCATCATCTCTAAGTTGAGAGGTAAGAAGTTCAAGTTGCCACCAGGTCCAACTGCTGTTCCAATCTTCGGTAACTGGTTGCAAGTTGGTGACGACTTGAACCACAGAAACTTGACTGACTTGGCTAAGAAGTTCGGTCAAATCTTCTTGTTGAGAATGGGTCAAAGAAACTTGGTTGTTGTTTCTTCTCCAGACTTGGCTAAGGAAGTTTTGCACACTCAAGGTGTTGAATTCGGTTCTAGAACTAGAAACGTTGTTTTCGACATCTTCACTGGTAAGGGTCAAGACATGGTTTTCACTGTTTACGGTGAACACTGGAGAAAGATGAGAAGAATCATGACTGTTCCATTCTTCACTAACAAGGTTGTTCAACAATACAGAAAGGGTTGGGAAGACGAAGCTGCTGCTGTTGTTGAAGACGTTAAGAAGAACCCAAAGTCTGCTACTGAAGGTGTTGTTATCAGAAAGAGATTGCAATTGATGATGTACAACAACATGTTCAGAATCATGTTCGACAGAAGATTCGACTCTGAAGACGACCCATTGTTCTTGAAGTTGAAGGCTTTGAACGGTGAAAGATCTAGATTGGCTCAATCTTTCGAATACAACTACGGTGACTTCATCCCAGTTTTGAGACCATTCTTGAGAGGTTACTTGAAGTTGTGTAAGGAAGTTAAGGACAAGAGATTGCAATTGTTCAAGGACTACTTCGTTGACGAAAGAAAGAAGATCGGTTCTACTAAGAAGTTGGACAACAACCAATTGAAGTGTGCTATCGACCACATCTTGGAAGCTAAGGACAAGGGTGAAATCAACGAAGACAACGTTTTGTACATCGTTGAAAACATCAACGTTGCTGCTATCGAAACTACTTTGTGGTCTATCGAATGGGGTATCGCTGAATTGGTTAACCACCCAGAAATCCAAGCTAAGTTGAGACACGAATTGGACACTAAGTTGGGTTCTGGTGTTCAAATCACTGAACCAGACGTTCAAAACTTGCCATACTTGCAAGCTGTTGTTAAGGAAACTTTGAGATTGAGAATGGCTATCCCATTGTTGGTTCCACACATGAACTTGCACGACGCTAAGTTGGGTGGTTTCGACATCCCAGCTGAATCTAAGATCTTGGTTAACGCTTGGTGGTTGGCTAACAACCCAGACCAATGGAAGAAGCCAGAAGAATTCAGACCAGAAAGATTCTTGGAAGAAGAAGCTAAGGTTGAAGCTAACGGTAACGACTTCAGATACTTGCCATTCGGTGTTGGTAGAAGATCTTGTCCAGGTATCATCTTGGCTTTGCCAATCTTGGGTATCACTTTGGGTAGATTGGTTCAAAACTTCGAATTGTTGCCACCACCAGGTCAATCTAAGATCGACACTTCTGAAAAGGGTGGTCAATTCTCTTTGCACATCTTGAAGCACTCTACTATCGTTGCTAAGCCAAGATCTTTCTAA |
| *4CL* | ATGGACTCTCAAAAGGAAATCATCTTCAGATCTAAGTTGCCAGACATCTACATCCCAAAGCACTTGCCATTGCACTCTTACTGTTTCGAAAACATCTCTAAGTTCTTGGACAGACCATGTTTGATCAACGGTGCTACTGGTGAAGTTCACACTTACGCTGACGTTGAATTGACTTCTAGAAAGGTTGCTTCTGCTTTGCACCAACAAGGTATCTCTAAGGGTGACGTTATCATGATCTTGTTGCCAAACTCTCCAGAATTCGTTTACTCTTTCATCGGTGCTTCTTACTTGGGTGCTATCTCTACTATGGCTAACCCATTCTTCACTGCTGCTGAAATCATCAAGCAAGTTAAGGCTTCTAACTCTAAGATCATCATCACTCAATCTGCTCACATCCCAAAGGTTAAGGACTACGCTTCTGACAACTCTATCAAGTTGGTTTGTATCGACTCTGCTCCATTGGGTTGTTTGCACTTCTCTGAATTGACTTCTGCTGACGAAACTAAGTTGCCACAAATCGAAGTTTCTTCTGACGACGTTGTTGCTTTGCCATACTCTTCTGGTACTACTGGTTTGCCAAAGGGTGTTATGTTGACTCACAAGGGTTTGGTTACTTCTGTTGCTCAACAAGTTGACGGTGAAAACCCAAACTTGTGGATACACTCTGAAGACGTTTTGATGTGTTCTTTGCCATTGTTCCACATCTACTCTTTGAACTCTATCTTGTTGTGTGGTTTGAGAGCTGGTGCTGCTATCTTGTTGATGTCTAAGTTCGACATCGTTCCATTCTTGCAATTGATCGAAAAGTACAAGGTTACTATCGGTCCATTCGTTCCACCAATCGTTTTGACTATCGCTAACAACGAAGAATTGGTTGACAAGTACGACATGTCTTCTATCAGAACTGTTATGTCTGGTGCTGCTCCATTGGGTAAGGACTTGGAAGACACTGTTAGAATGAAGTTCCCAAACGCTAAGTTGGGTCAAGGTTACGGTATGACTGAAGCTGGTCCAGTTTTGGCTATGTGTTTGGCTTTCGCTAAGGAACCATTCGACATCAAGTCTGGTGCTTGTGGTACTGTTGTTAGAAACGCTGAAATGAAGATCGTTGACCCAGACTCTGGTGTTTCTTTGCCAAGAAACCAAAGAGGTGAAATCTGTATCAGAGGTGACCAAATCATGAAGGGTTACTTGAACGACCCAGAAGCTACTAAGAGAACTATCGACTCTGAAGGTTGGTTGCACACTGGTGACATCGGTTTGATCGACGACGACGACGAATTGTTCATCGTTGACAGATTGAAGGAATTGATCAAGTACAAGGGTTTCCAAGTTGCTCCAGCTGAATTGGAAGCTTTGTTGTTGACTCACCCAGACATCTCTGACGCTGCTGTTGTTCCAATGATCAACGAAGCTGCTGGTGAAGTTCCAGTTGCTTTCGTTGTTAAGACTAACGGTTCTTCTGTTACTGAAGACGACATCAAGCAATTCGTTTCTAAGCAAGTTGTTTTCTACAAGAGAATCAACAGAGTTTTCTTCTGTGAAACTATCCCAAAGTCTCCATCTGGTAAGATCTTGAGAAAGGACTTGAGAGCTAAGTTGGCTGCTGGTGTTCCATCTTAA |
| *CHS* | TTAAGTAGCGATAGCAGTAGTAGTTGGCAAAGAGTGCAAAACAACAGTTTCAACAGTCAAACCTGGACCGAAACCGAACAAAACACCCCAGTCCAAACCTTCACCAGTAGTAGCAGCACCGTCTTCAGCAGACTTCTTTCTCATTTCGTCGATGATGAACAAAACACAAGCAGAAGACATGTTACCGTATTCAGACAAAACGTGTCTAGTAGCTCTCATCTTTTCTTCCTTCAAACCCAACTTCAATTCAACTTGGTCCAAGATAGCTGGACCACCTGGGTGAGCGATCCAGAACAAAGAGTTCCAGTCAGAGATACCCAATGGAGAGAAAGCTTGAGTCAAAGCCTTTTCGATGTTCTTAGAGATCAAACCTGGAACGTCCTTCAACAAGTGGAAAGTCAAACCAACTTCTCTCAAGTGACCGTCGATAGCACCTTCAGAGTCTGGCAAGATAGTTTGAGCAGCAGAGATCATTTCGAACAATGGTCTTTCAGTAGTCAAGTCTGGGTCAGAACCAACGATAACAGCAGCAGCACCGTCACCGAACAAAGCTTGACCAACCAAAGAGTCCAAGTGAGTGTCGTTTGGACCTCTGAAAGTAACAGCAGTGATTTCAGAACAAACAACCAAAACTCTAGCACCCTTGTTGTTTTCAGCCAAGTCCTTAGCCAATCTCAAAACAGTACCACCAGCGAAACAACCTTGTTGGTACATCATGAATCTCTTAACAGATGGTCTCAAACCCAACAACTTAGTCAATTGGTAGTCAGCACCTGGCATGTCAACACCAGAAGTAGTACAGAAGATCAAGTGAGTGATCTTAGACTTTGGTTGACCCCATTCCTTGATAGCCTTAGTAGCAGCTTCCTTACCCAACTTTGGAACTTCAACAACAACAACGTCTTGTCTAGCGTCCAAAGATGGAGCCATGTATTCACACAAAGATGGGTTTTCCTTCAAGTATTCTTCAGTCAAGTGCATGTATCTCTTTCTGATCATAGACTTGTCACACATTCTCTTGAACTTTTCCTTCAAGTCAACCATGTGTTCAGACTTAGTGATTCTGAAGTAGTAGTCTGGGTAGTCAGCTTGGTAAACACAGTTAGATGGAGTAGCAGTACCGATAGCCAAGATAGTAGCTGGACCTTGAGCTCTTTGAGCTTCTCTGATAGCAGCGATGTCGATAGAAGAAGCCAT |
| *CHI* | ATGGCTGCTACTACTACTCCATTGACTACTTCTTTGCAAGTTGAATCTATCGTTTTCCCATCTTCTGTTAAGCCACCAGGTTCTACTAAGTCTTTGTTCTTGGGTGGTGCTGGTGTTAGAGGTATGGAAATCCAAGGTAACTTCGTTAAGTTCACTGGTATCGGTGTTTACTTGGAAGACAAGGCTATCCCATTGTTGGCTGCTAAGTGGATGGGTAAGTCTTCTACTGAATTGTTGGACTCTGTTGAATTCTTCAGAGACATCGTTACTGGTCCATTCGAAAAGTTCACTCAAGTTACTATGATCTTGCCATTGACTGGTAAGCAATACTCTGAAAAGGTTTCTGAAATGTGTGTTGGTGTTTGGAAGGCTCACGGTGTTTACACTGACGCTGACGGTACTACTATCGAAAAGTTCTTGGAAGTTTTCAAGGACAAGAACTTCTTGCCAGGTTCTTCTATCTTGTTCACTACTTCTCCATTGGGTTCTTTGACTATCTCTTTCTCTAAGGACTCTACTATCCCAGAAGCTGCTAACGTTGTTTTGGAAAACGAAAAGTTGTCTCAAGCTGTTATCGAATCTGTTATCGGTAAGAACGGTGTTTCTCCAGCTACTAAGCAATCTTTGGCTTCTAGATTGTTCGACTTGATGAAGAAGTTCGACGAAGAATTGTCTGCTTCTGTTGAAGTTGCTGACGTTTCTAAGTACGGTTTGTAA |
| Ter-4  (originally designed sequence) | ggatccCGTCTCACCAGACGATACAGAGGCTAAGAATAACGCAGATAATCGCTCTAACGAAACGTACTAAAAGATTTCTTTTGAAGTAACTAGATACCCTGGTCTTATACTAGGTATCTTTGTCAGAAACGGCCTAAGACTACAGTAAGAGCAGTTGGAACCTggtaccTAAAATTTTGATCTATTGTAGTCGCCTAATCTTGCTTCTCTTGCTGAATAAATAGCGCTATCCGCTGTACCGTACCAAGAGTGACCTACAGCGTTTGATAGGCAAAAGAGTTAAGGACAGACTGGGTTTCAATCAAAATAAATGCCAAAAGGATTTGACTTTATATTTGATAATGCTCACTCTAAACGGAAGGCACATGCATCGCATAATTGGAACGAGAAATAAAGTGTTATTATAAGTGTACAAAATTATTTAATGGTTGATTATAAAAAACTATTTTATAATTTGTAGACAACAAGCaagcttAGGTtgagacccccggggagagtcgacctgcagggtctcaATCGgaattcATAAAGCAATCTTGATGAGGATAATGATTTTTTTTTGAATATACATAAATACTACCGTTTTTCTGCTAGATTTTGTGAAGACGTAAATAAGTACATATTACTTTTTAAGCCAAGACAAGATTAAGCATTAACTTTACCCTTTTCTCTTCTAAGTTTCAATACTAGTTATCACTGTTTAAAAGTTATGGCGAGAACGTCGGCGGTTAAAATATATTACCCTGAACGTGGTGAATTGAAGTTCTAGGATGGTTTAAAGATTTTTCCTTTTTGGGAAATAAGTAAACAATATATTGCTGCCTTgagctcCAGCCAACCAGTCAGATTAGCAGTATATAGGCTGCGAGTTACTAGCGGGCAAAATCTGGAGTTATCGCCTAAAGCGTTATCTGATCCCTATTCGGCAGCAGTTTGGTCGGTTTGAGTAGTCAATAACGTTCTATCGTATCTCGTTCGTCGAGAGACGtctaga |

**Table S4. Primers used in this work**

| **Name** | **Sequence** |
| --- | --- |
| T4-YCP-F | CACACAGGAAACAGCTATGACTGTAAAACGACGGCCAGTGAA |
| T4-YCP-R | GTTGTAAAACGACGGCCAGTTGAGCGGATAACAATTTCACACAG |
| Y22-T4-F | GTCATAGCTGTTTCCTGTGTGAAATTG |
| Y22-T4-R | ACTGGCCGTCGTTTTACAACGTC |
| P1-F | CTGGTCTCACATCTTTGTTTGTTTATGTGTGTTTATTCGA |
| P1-R | CTGGTCTCAATTCTGTATATGAGATAGTTGATTGTATGCT |
| AtF3H-GG2-F | CCCGGTCTCAGAATGGCTCCAGGAACTTTGA |
| AtF3H-GG2-R | CCCGGTCTCAAGGTTAAGCGAAGATTTGGTCGA |
| AtFLS-GG1-F | CCCGGTCTCAGATGGAGGTCGAAAGAGTCCAA |
| AtFLS-GG1-R | CCCGGTCTCACGATTCAATCCAGAGGAAGTTTATTGAGCT |
| CitFLS-GG1-F | CCCGGTCTCAGATGGAAGTTGAACGCGTTC |
| CitFLS-GG1-R | CCCGGTCTCACGATTTACTGCGGCAGTTTGTTCAG |
| MdFLS-GG1-F | CCCGGTCTCAGATGGGTGTTGAAAGCGTTGA |
| MdFLS-GG1-R | CCCGGTCTCACGATTTACTGCGGCAGTTTGTTGA |
| PeFLS-GG1-F | CCCGGTCTCAGATGGAGTTCGACAGAGTTCA |
| PeFLS-GG1-R | CCCGGTCTCACGATTTACTGTGGCAACCTGTT |
| ZmFLS-GG1-F | CCCGGTCTCAGATGGGTGGTGAAACCCATCT |
| ZmFLS-GG1-R | CCCGGTCTCACGATTTACATCGGCAGTTTGTTGAT |
| F3H-CPF | CACACAGGAAACAGCTATGACTATACATGTAGGTGGCGGA |
| F3H-CPR | GTTGTAAAACGACGGCCAGTTAAGAGCAGTTGGAACCTG |
| Y33-F | GAATTCACTGGCCGTCGTTTTACAACGTCG |
| Y33-R | AAGCTTGGCGTAATCATGGTCATAGCTGTT |
| ALAC-GibF | GACCATGATTACGCCAAGCTTACAGTAAGAGCAGTTGGAACCT |
| ALAC-GibR | GCTAATCTGACTGGTTGGCTG |
| ADH2-GibF | AGCCAACCAGTCAGATTAGCAGTGGCAGCACGCTAATTC |
| ADH2-GibR | ACGGCCAGTGAATTCGAGCTCTGTCAGGTAGAACTCGTCCAAT |
| ACAD-GibF | CAGCCAACCAGTCAGATTAGCA |


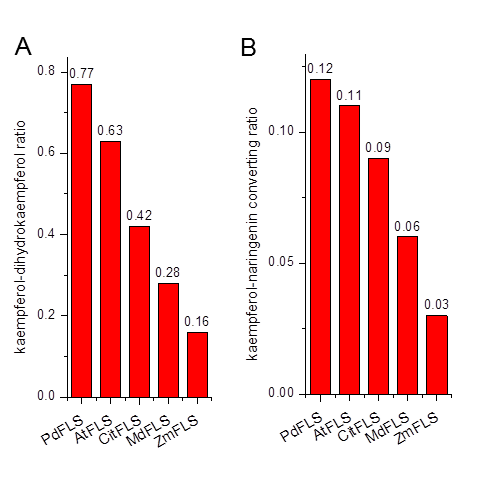


**Figure S1. Mole ratio of kaempferol to dihydrokaempferol (A) and mole ratio of kaempferol to total naringenin (B) in whole cell catalysis with the recombinants expressing FLS orthologs.**


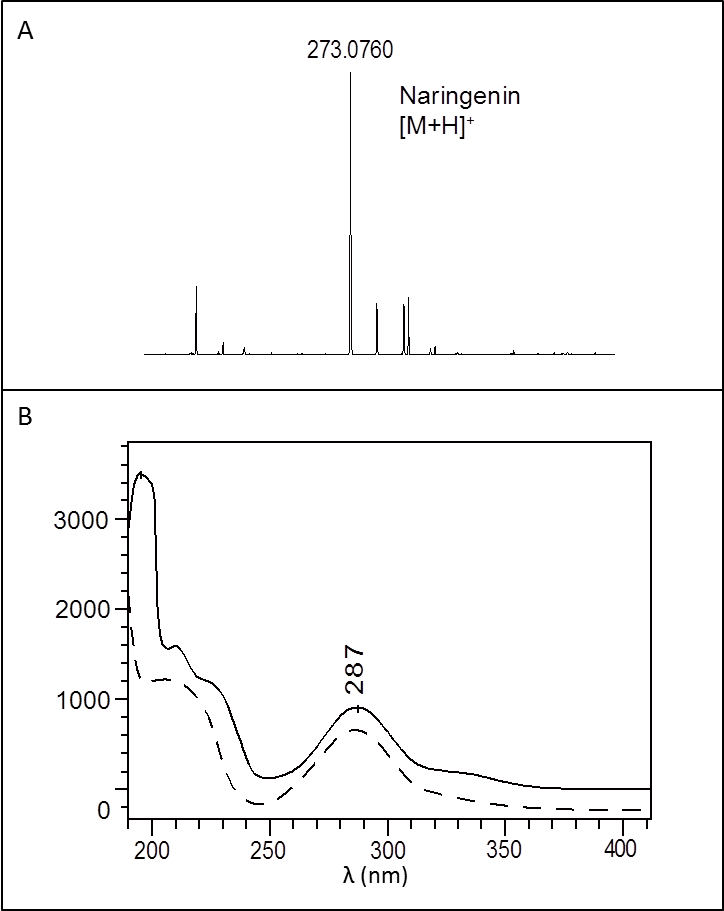


**Figure S2. Identification of naringenin produced by W3NP from glucose.**

A, LC-MS analysis of naringenin produced by W3NP. B, UV absorption of naringenin produced by W3NP (solid line) and standard (dash line).


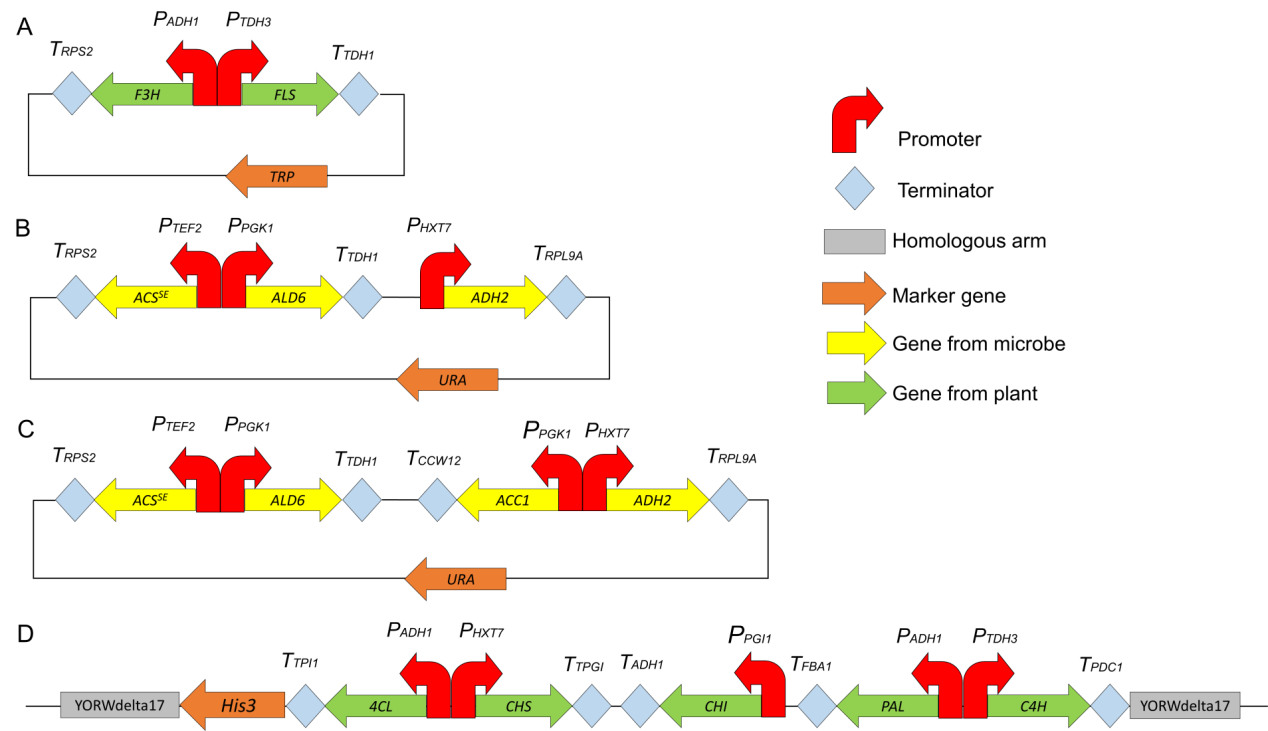


**Figure S3. Scheme of plasmids construction and gene manipulation for kaempferol production**

A, Sketch drawing for F3H and FLS expression. B, Y33-ALAC-ADH2, a plasmid expressing *ALD6*, *ACS^SE^* and *ADH2*. C, Y33-ALAC-ACAD, a plasmid expressing *ALD6*, *ACS^SE^*, *ADH2* and *ACC1*. D, Genome manipulation in W3NP for naringenin production.


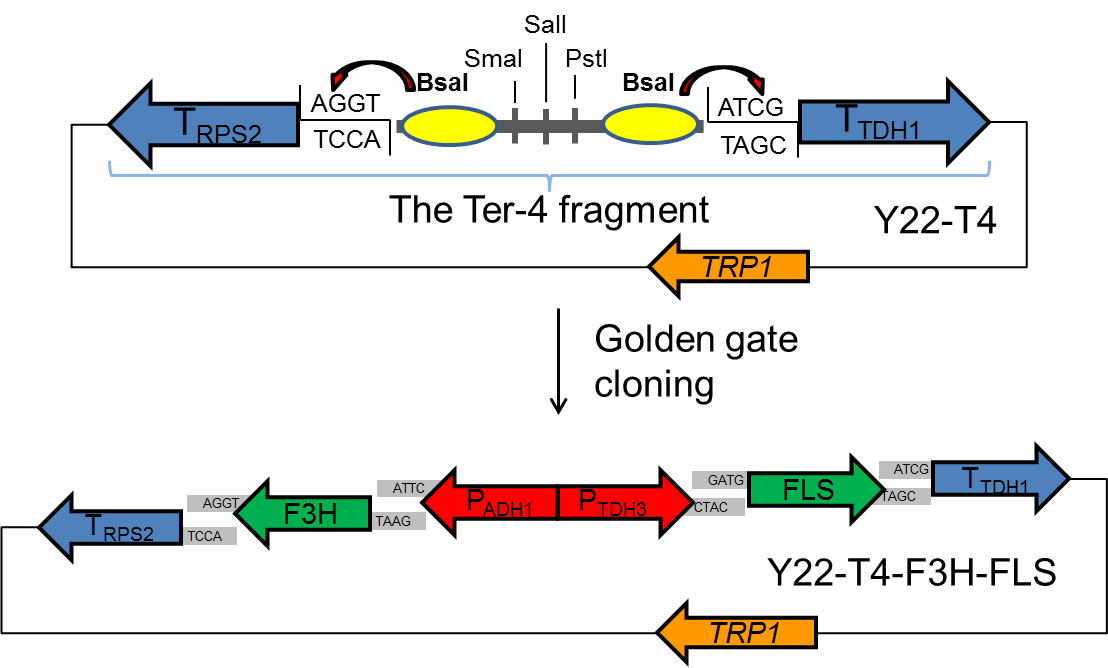


**Figure S4. Scheme of cassette assembly through golden gate cloning.**

A. The design of Ter-4 fragment and the cloning vector Y22-T4. The vector Y22-T4, DNA fragments of promoter, F3H and FLS were digested by *Bsa*I and ligated through sticky ends
